# Spousal Concordance of Physical Frailty in Older Korean Couples

**DOI:** 10.3390/ijerph17124574

**Published:** 2020-06-25

**Authors:** Suah Kang, Miji Kim, Chang Won Won

**Affiliations:** 1 Department of Medicine, College of Medicine, Kyung Hee University, Seoul 02447, Korea; skang39@khu.ac.kr; 2 Department of Biomedical Science and Technology, College of Medicine, East-West Medical Research Institute, Kyung Hee University, Seoul 02447, Korea; 3 Elderly Frailty Research Center, Department of Family Medicine, College of Medicine, Kyung Hee University, Seoul 02447, Korea

**Keywords:** frailty, spousal concordance, aging

## Abstract

Marital status is an important risk factor for physical frailty. However, there are limited data on spousal concordance of physical frailty among married couples. Here, we evaluate the spousal concordance of frailty as defined by the Fried frailty phenotype and specific phenotype components that contribute to this association. Data on 315 married couples (630 individuals) aged between 70 and 84 years were obtained from the Korean Frailty and Aging Cohort Study (KFACS). Multivariate logistic regressions were used for the analysis. After adjusting for covariates (age, body mass index, education, house ownership, comorbidity, cognition, depressive symptoms, cohabitation with adult children for both partners), a husband’s frailty was positively associated with his wife’s frailty (odds ratio (OR) 3.34, 95% confidence interval (CI) 1.04–10.73, *p* < 0.05), and a wife’s frailty was significantly associated with her husband’s frailty (OR 4.62, 95% CI 1.31–16.33, *p* < 0.05), indicating a greater effect of the frailty status of the spouse among women than among men. Among the five components of the Fried frailty phenotype, weight loss, slowness, and exhaustion were the main contributing factors to the spousal association for frailty. In conclusion, having a frail spouse is a strong and independent risk factor for frailty among community-living older adults.

## 1. Introduction

The aged population is growing rapidly. By 2050, the proportion of the population over 60 years old will almost double from 12% to 22%, reaching up to 434 million worldwide [1]. Frailty has become an area of interest for those concerned with healthy aging [2,3]. Frailty is a state of functional decline and increased vulnerability, commonly defined by the Fried phenotype model and the frailty index [4,5,6]. The Fried phenotype model is a physical frailty criterion, and the frailty index is a cumulative health deficits index including physical, psychological, mental, and social functions [4,6]. In community-dwelling adults aged ≥65 years, the estimated prevalence of frailty is around 10%, and the incidence may increase with age [7,8]. Frailty may have a high health care burden since it is associated with increased morbidity and mortality in the elderly [9,10].

Frailty is associated with sociodemographic, physical, biological, lifestyle, and psychological risk factors [11]. A recent study identified marital status as an important risk factor for physical frailty [12]. Most caregivers of frail older adults are their spouses [13,14,15]. Spousal caregivers experience reduced quality of life because of their restricted participation in daily activities [16]. There are negative impacts on the physical and mental health of spousal caregivers [17]. Furthermore, in a case–control study, frailty status worsened in one-third of the spousal caregivers of older patients with cognitive and functional impairment [18]. However, limited studies have examined the spousal correlation of frailty among community-living older adults. One study found a positive correlation between an individual’s frailty and the spouse’s frailty [19]. However, the study failed to control for the major risk factors for frailty, including comorbid conditions, cognitive impairment, and depression. It is unclear to what extent having a frail spouse contributes to one’s frailty status after adjusting for these risk factors. In this study, we evaluated the spousal concordance of frailty among older adults and determined the specific frailty phenotype components that contribute to this association.

## 2. Materials and Methods

### 2.1. Participants

Data were mainly derived from the Korean Frailty and Aging Cohort Study (KFACS) 2016 and 2017 baseline database. The KFACS is a 2-year, nationwide, multicenter, prospective cohort study to identify risk factors and outcomes of frailty and develop interventions for prevention and management. The KFACS recruited 3014 community-living elderly individuals aged between 70 and 84 years in 2016 and 2017. The participants were recruited from 10 study centers across different regions and different residential locations (urban, suburban, and rural) [20]. Each center recruited participants using quota sampling stratified by age (70–74, 75–79, and 80–84 years with a ratio of 6:5:4) and sex (male and female with same ratio) [20]. Participants were recruited from diverse settings (local senior welfare centers, community health centers, apartments, housing complexes, and outpatient clinics) to minimize selection bias [20]. The inclusion criteria for KFACS participants were as follows: aged 70–84 years, currently living in the community, having no plans to move out in the next 2 years, and having no problems with communication and no prior dementia diagnosis [20]. In this case, “move out” refers to relocating to areas outside the three neighboring towns [20]. Exclusion criteria included individuals with difficulty in giving opinion, those unable to comply with the study requirements, or those deemed inappropriate on the basis of the findings or evaluation by the researcher. The detailed study design has been published previously [20]. Spousal pairs were identified by marital status, cohabitation status, and home address. Subjects were considered spouses if both male and female adults reported being married, currently lived with their spouse, and had the same home address. A total of 333 samples of married couples were confirmed to be eligible for the present study. The final analysis included 315 married couples, after excluding 18 with missing data, for assessing the Fried physical frailty phenotype. The Clinical Research Ethics Committee of the Kyung Hee University Hospital approved the KFACS protocol (institutional review board (IRB) number 2015-12-103). The present study was exempt from the requirement for IRB approval by the Clinical Research Ethics Committee of the Kyung Hee University Medical Center (IRB number: 2020-05-066).

### 2.2. Frailty Assessment

The primary outcome of this study was the frailty status. Frailty was defined by the Fried frailty phenotype. The Fried frailty phenotype comprises five criteria, namely weight loss, weakness, exhaustion, slowness, and low physical activity [4]. Those satisfying ≥3 components were considered frail. Weight loss was defined as an unintentional weight loss of ≥10 pounds. Weakness was defined as a baseline grip strength in the lower 20%, adjusted for sex and body mass index (BMI) in the KFACS population distribution. Exhaustion was assessed by the responses to two statements from the Center for Epidemiologic Studies Depression Scale: (1)” I felt that everything I did was an effort”, and (2) “I could not get going”. Slowness was defined as the slowest 20% at baseline on the basis of the 4-m usual gait speed, adjusted for sex and height in the KFACS population distribution. Low physical activity was defined as the lower 20% of sex-specific kcals per week, calculated on the basis of the International Physical Activity Questionnaire in a general Korean population-based survey of older adults [21].

### 2.3. Covariates

Age, BMI, education, house ownership, comorbidity, cognition, depressive symptoms, and cohabitation with adult children of both husband and wife were used as covariates in this study. Data on demographics, including age, BMI, education, house ownership, and cohabitation with adult children were self-reported. Comorbidity was defined as having ≥2 comorbid conditions. Cognitive impairment was assessed using the Mini-Mental State Examination (MMSE). Depressive symptoms were assessed using the short version of the Geriatric Depression Scale. Nutritional status was determined using the Korean version of the short form of the Mini Nutritional Assessment (MNA-SF).

### 2.4. Statistical Analysis

All analyses were conducted using SPSS (ver. 25.0; IBM Corp., Armonk, NY, USA). A comparison of characteristics by sex was performed using the chi-squared test for categorical variables and the independent *t*-test for continuous variables. The spousal association of frailty and the five components of the Fried frailty phenotype were analyzed using multivariate logistic regression models and described by odds ratios (ORs) with 95% confidence intervals (CIs). Four models were considered in addition to the unadjusted model: (1) adjusted for age; (2) additionally adjusted for BMI, education, house ownership, comorbidity, and cognition; (3) additionally adjusted for depressive symptoms; and (4) additionally adjusted for cohabitation with adult children of both partners in all analyses. A two-sided *p* < 0.05 was considered statistically significant.

## 3. Results

The demographic and health characteristics of the study population (630 subjects, or 315 married couples) are listed in Table 1. The mean ages of husbands and wives were 77.6 ± 3.4 and 74.7 ± 3.6 years, respectively. Husbands had a lower mean BMI (23.7 ± 3.0 kg/m^2^) and higher education status (77.1%) than wives. Wives were more likely to have house ownership (31.1%). There was no significant difference in the current employment status. Wives had higher prevalence of cognitive impairment (24.1%), comorbidities (61.2%), and depressive symptoms (23.5%) than husbands. In addition, the demographic and health characteristics of the frail and nonfrail groups of study participants are presented in Appendix A.

Table 2 shows the frailty status determined by the Fried frailty phenotype and its five components by sex. Subjects classified as robust or prefrail in the Fried frailty phenotype were considered nonfrail. Frailty was more prevalent among husbands than wives; 35 (11.1%) husbands and 28 (8.9%) wives were classified as frail. The percentages of all five components except exhaustion were higher in husbands.

Table 3 and Table 4 summarize the multivariate logistic regression models examining the spousal association of frailty and for the five components of the Fried frailty phenotype. The wife’s frailty was significantly associated with the frailty of her husband in all models (OR 3.13–4.76, *p* < 0.05). Similarly, the husband’s frailty was positively associated with the frailty of his wife in all models (OR 2.91–4.76, *p* < 0.05) except model 3 (OR 3.19, 95% CI 1.00–10.22, *p* = 0.05). After adjusting for possible covariates, a frail husband had 4.62 odds of having a frail wife, and a frail wife had 3.34 odds of having a frail husband (model 4). While adjusting for other covariates reduced the odds ratio, adjusting for depressive symptoms (model 3) and cohabitation with adult children (model 4) increased the odds ratio.

Among the five components of the Fried frailty phenotype, there was an association between the husband and wife in regard to weight loss, exhaustion, and slowness. Weight loss in one spouse was significantly associated with the increased risk of weight loss in the partner in all models (husband, OR 6.89–8.56, *p* < 0.01; wife, OR 4.91–6.95, *p* < 0.05). After adjusting for covariates, wives with weight loss had 8.34 odds of having a husband with weight loss (*p* < 0.01). A husband with weight loss had 4.91 odds of having a wife with weight loss (*p* < 0.01). Exhaustion in one spouse was also associated with greater risk of exhaustion in the partner in all models (husband, OR 2.00–2.23, *p* < 0.05; wife, OR 1.94–2.23, *p* < 0.05). Slowness in one spouse was associated with higher odds of slowness in the partner in all models (husband, OR 2.50–2.82, *p* < 0.05; wife, OR 2.52–2.82, *p* < 0.05) except model 2 (husband, OR 1.87, 95% CI 0.95–2.51; wife, OR 1.88, 95% CI 0.95–3.72). There was no significant association between spouses for low activity and muscle weakness.

## 4. Discussion

In this study, we found that older adults with frail spouses had significantly higher odds of being frail. Husbands and wives with a frail partner had 3.34 and 4.62 times higher odds of being frail, respectively, than if their partner was not frail. Thus, women were more affected by spousal frailty status than men. Among the five components of the Fried frailty phenotype, there was a spousal association in regard to weight loss, slowness, and exhaustion. The odds ratio for a wife to experience weight loss if her husband had weight loss was twice the odds ratio for weight loss in a husband if his wife had weight loss. These results suggest the presence of sex differences in our findings.

We found a spousal concordance for frailty among community-living older adults. This finding is consistent with the results from a previous study that reported that frailty in one spouse is related to greater subsequent frailty in the other [19]. It is also consistent with the previously reported concordance of physical, functional, and mental health and health behavior change in married couples [22,23,24,25]. Concordant health decline, especially in an elderly couple, is a significant risk factor for difficulties in physical activities, functional disabilities, and depressive symptoms that have an increased caregiver burden [26]. Thus, spousal frailty can be used to detect, prevent, and manage a couple’s frailty among community-living older adults.

Our study suggests an independent association between frailty status of marital partners in the older population. The logistic regression models were adjusted for recognized frailty-related factors, including age, education, cognition, comorbidity, and depression [27]. The significant association for frailty within married couples remained after the adjustment for these factors. Additionally, the adjustment for the covariates not only decreased but also increased the size of the odds ratios. The change in the direction of the association may be due to the interplay of positive and negative confounding [28,29]. Further research is needed to examine the relationship between these factors, especially the cohabitation with adult children, as its impact on frailty has not been fully investigated. In previous studies, concordance of mood (depressive symptoms, neuroticism) [30,31], social activity [32], chronic conditions [23,33,34], and cognitive decline [35] between married couples have been observed. The multifactorial nature of spousal association and frailty may explain the varying strength of the spousal association.

We identified that three components of the Fried frailty phenotype, namely, weight loss, slowness, and exhaustion, mainly contribute to the spousal association for frailty. The strength of the spousal association was strongest for weight loss in both husbands and wives. There are various causes of weight loss among the elderly, and they often coexist. Common causes are classified as organic (neoplastic, nonneoplastic, and age-related physiological changes), psychological (depression, dementia, and anxiety disorders), nonmedical (socioeconomic conditions such as poverty), and unknown [36,37]. These conditions can be directly (such as environmental factors) and indirectly (such as depression [31]) shared by married couples. Furthermore, activities related to food preparation are mainly performed by women rather than men, especially among older adults in Korea [38]. The influence of gender role needs is discussed later.

Exhaustion is associated with the psychosocial condition of older adults. Older adults can be affected by the depressive symptoms and poor physical health of their spouses [39]. Those living with a depressed spouse are more likely to experience depressive mood or episodes [40,41], contributing to the increased association for exhaustion among married couples. A recent study suggested an association between slow gait speed and social networks among older adults [42]. Thus, the sharing of a couple’s social network may influence the spousal concordance in slowness.

We found that women were more affected by the frailty status and weight loss of their spouses than men. This finding may be due to gender role orientation and socialization [43]. These perspectives are deeply ingrained in the minds of the Korean elderly and are widely reflected in their daily lives. According to the 2017 national survey of living conditions and welfare needs of the Korean elderly, 93.7% of male adults aged >65 years received instrumental support from their spouse compared to only 54% of females aged >65 years [44]. Similarly, men were less likely to provide and more likely to receive caregiving than women [44]. Poor spousal health, low education, and unemployment decreased the rate of receiving instrumental support and caregiving [44]. Thus, it can be deduced that elderly women do more housework and have a higher caregiving burden than men [45]. Furthermore, female caregivers are more likely to experience physical and psychosocial distress [46,47,48]. Our findings are in agreement with women’s vulnerability to spousal influence and its negative health effects as reported in previous studies on cognitive functioning [35], vision impairment [49], and depressive symptoms [50].

There are limitations to our study. First, due to the cross-sectional study design, it is difficult to understand the causal relationship from our analysis. Further longitudinal studies are needed to evaluate the temporal changes in the frailty status in married couples. Second, as the KFACS was designed to target the elderly population regardless of marital status, the sample size of married couples was relatively small. However, our results suggest a similar prevalence of frailty and concordant frailty within couples as reported in previous studies [19]. In addition, the quality of the marital relationship was not accounted for in our study.

## 5. Conclusions

In conclusion, having a frail spouse is a strong and independent risk factor for frailty. Among the five components of the Fried frailty phenotype, weight loss, exhaustion, and slowness were associated within married couples. Weight loss had the strongest association. Knowledge of the spousal association for frailty can help to prevent spousal frailty by managing risk factors and developing interventions designed for older married couples. Further prospective and longitudinal investigations will help corroborate our findings.

## Figures and Tables

**Table 1:** Demographic and health characteristics of study participants.

Variables	Husband (*n* = 315)	Wife (*n* = 315)	*p*-Value
**Demographics**			
Age, mean (SD), year	77.6 (3.4)	74.7 (3.6)	<0.001
BMI, mean (SD), kg/m^2^	23.7 (3.0)	24.7 (2.8)	<0.001
Education, *n* (%)			
Less than 7 years	72 (22.9)	168 (53.3)	<0.001
Residence, *n* * (%)			
Urban	80 (25.6)	80 (25.6)	1.000
Suburban	148 (47.4)	148 (47.4)	
Rural	84 (26.9)	84 (26.9)	
House ownership, *n* (%)	30 (9.5)	98 (31.1)	<0.001
Currently employed, *n* * (%)	81 (25.8)	68 (21.7)	0.223
Living with adult children, *n* * (%)	64 (20.6)	64 (20.6)	
**Health Characteristics**			
Cognitive Impairment			
MMSE score <24	46 (14.6)	76 (24.1)	0.002
Health Behavior			
Current smoker, *n* (%)	33 (10.5)	0 (0.0)	<0.001
Heavy drinking, *n* (%)	106 (33.7)	10 (3.0)	<0.001
Comorbid Conditions			
Number of comorbid conditions, mean (SD)	1.46 (1.24)	1.99 (1.36)	<0.001
Comorbidity, *n* (%)	141 (42.7)	202 (61.2)	<0.001
Hypertension, *n* (%)	174 (55.2)	174 (55.2)	1.000
Diabetes mellitus, *n* (%)	77 (24.4)	69 (21.9)	0.450
Mental Health			
Depressive symptom (GDS score ≥6), yes, *n* (%)	49 (15.6)	74 (23.5)	0.012
Physical Activity			
Moderate/vigorous activity, none, *n* (%)	107 (34.0)	116 (36.8)	0.453
Social Activity			
Religious meeting, none, *n* * (%)	155 (49.4)	93 (29.6)	<0.001
Social meeting, none, *n* (%)	72 (22.9)	108 (34.3)	0.001
Possible Malnutrition			
MNA score ≤11, *n* * (%)	30 (9.6)	19 (6.1)	0.102
Self-perceived health, poor, *n* (%)	81 (25.7)	119 (37.8)	0.001

Notes: Comorbidity: ≥2 comorbid conditions; BMI = body mass index; GDS = Geriatric Depression Scale; MMSE = Mini-Mental State Examination; MNA = Mini Nutritional Assessment. Chi-squared test for categorical variables and independent *t*-test for continuous variables. *n* * indicates missing data.

**Table 2:** Proportion of Fried frailty phenotype by sex.

Variable	Husband (*n* = 315)	Wife (*n* = 315)	Both (*n* = 315 Pairs)	*p*-Value
	Total, *n* (%)	Total, *n* (%)	Total, *n* (%)	
**Fried Frailty Phenotype**				
Frail	35 (11.1)	28 (8.9)	9 (2.9)	0.353
**Five Components of Frailty Phenotype**				
Weight loss	22 (7.0)	17 (5.4)	5 (1.6)	0.408
Exhaustion	77 (24.4)	133 (42.2)	44 (14.0)	<0.001
Low activity	42 (13.3)	25 (7.9)	6 (1.9)	0.028
Weakness	74 (23.5)	56 (17.8)	17 (5.4)	0.076
Slowness	79 (25.0)	53 (16.8)	23 (7.3)	0.011

Notes: *p*-values were determined using the chi-squared test for categorical variables.

**Table 3:** Husband’s odds of frailty when the wife is frail in 315 married couples.

Frailty of Wife	Frailty of Husband (*n* = 315)				
	Unadjusted	Model 1	Model 2	Model 3	Model 4
	OR (95% CI)				
**Frailty of Wife**					
Frail wife	4.76 (1.95–11.58) ***	3.86 (1.50–9.92) **	2.91 (1.04–8.16) *	3.19 (1.00–10.22)	3.34 (1.04–10.73) *
**Five Components of Frailty Phenotype among Wives**					
Weight loss	6.89 (2.18–21.80) ***	6.95 (2.10–23.02) ***	6.87 (1.71–27.64) **	5.03 (1.14–22.23) *	4.91 (1.10–21.97) *
Exhaustion	2.23 (1.32–3.76) ***	2.18 (1.28–3.70) ***	1.94 (1.10–3.42) *	2.09 (1.09–3.98) *	2.14 (1.11–4.101) *
Low activity	2.23 (0.83–5.95)	1.66 (0.60–4.62)	1.85 (0.59–5.78)	2.00 (0.60–6.63)	2.12 (0.63–7.08)
Weakness	1.54 (0.81–2.93)	1.30 (0.67–2.52)	1.05 (0.51–2.15)	1.04 (0.50–2.18)	1.02 (0.49–2.13)
Slowness	2.82 (1.52–5.23) ***	1.88 (0.95–3.72)	2.55 (1.17–5.59) *	2.58 (1.16–5.70) *	2.75 (1.23–6.14) *

Notes: * *p* < 0.05, ** *p* < 0.01, *** *p* < 0.005. BMI = body mass index; CI = confidence interval; OR = odds ratio. Model 1 was adjusted for age; model 2 was adjusted for variables in model 1 plus BMI, education, house ownership, comorbidity, and cognition; model 3 was adjusted for variables in model 2 plus depressive symptoms; and model 4 was adjusted for cohabitation with adult children plus model 3.

**Table 4:** Wife’s odds of frailty when the husband is frail in 315 married couples.

Frailty of Husband	Frailty of Wife (*n* = 315)				
	Unadjusted	Model 1	Model 2	Model 3	Model 4
	OR (95% CI)				
**Frailty of Husband**					
Frail husband	4.76 (1.95–11.58) ***	3.96 (1.52–10.33) ***	3.13 (1.06–9.27) *	4.10 (1.16–14.56) *	4.62 (1.31–16.33) *
**Five Components of Frailty Phenotype among Husbands**					
Weight loss	6.89 (2.18–21.80) ***	7.07 (2.13–23.47) ***	8.47 (2.02–35.52) ***	8.56 (1.76–41.65) **	8.34 (1.70–40.85) **
Exhaustion	2.23 (1.32–3.76) ***	2.17 (1.27–3.68) ***	2.00 (1.13–3.57) *	2.11 (1.10–4.05) *	2.17 (1.12–4.22) *
Low activity	2.23 (0.83–5.95)	1.75 (0.63–4.83)	2.02 (0.63–6.48)	2.46 (0.72–8.37)	2.49 (0.72–8.55)
Weakness	1.54 (0.81–2.93)	1.29 (0.67–2.51)	1.04 (0.50–2.15)	1.03 (0.49–2.19)	1.03 (0.49–2.19)
Slowness	2.82 (1.52–5.23) ***	1.87 (0.95–3.69)	2.50 (1.13–5.53) *	2.48 (1.12–5.50) *	2.77 (1.24–6.31) *

Notes: * *p* < 0.05, ** *p* < 0.01, *** *p* < 0.005. BMI = body mass index; CI = confidence interval; OR = odds ratio. Model 1 was adjusted for age; model 2 was adjusted for variables in model 1 plus BMI, education, house ownership, comorbidity, and cognition; model 3 was adjusted for variables in model 2 plus depressive symptoms; and model 4 was adjusted for cohabitation with adult children plus model 3.

## Appendix A

**Table A1:** Demographic and health characteristics of frail and nonfrail groups.

Variables	Frail (*n* = 63)	Nonfrail (*n* = 567)	*p*-Value
**Demographics**			
Sex			
Male	35 (5.6)	280 (44.4)	0.864
Female	28 (4.4)	287 (45.6)	
Age, mean (SD), year	78.2 (3.4)	75.9 (3.7)	<0.001
BMI, mean (SD), kg/m^2^	24.0 (3.2)	24.2 (2.9)	0.551
Education, *n* (%)			
Less than 7 years	40 (63.5)	200 (35.3)	<0.001
Residence, *n* * (%)			
Urban	4 (27.7)	4 (6.5)	<0.001
Suburban	32 (51.6)	265 (47.0)	
Rural	26 (41.9)	143 (25.4)	
House ownership, *n* (%)	53 (84.1)	514 (90.7)	<0.001
Currently employed, *n* * (%)	11 (17.5)	138 (24.4)	0.220
Living with adult children, *n* * (%)	10 (15.9)	121 (21.4)	0.026
**Health Characteristics**			
Cognitive Impairment			
MMSE score <24	27 (42.9)	95 (16.8)	<0.001
Health Behavior			
Current smoker, *n* (%)	4 (6.3)	29 (5.1)	0.676
Heavy drinking, *n* (%)	12 (19.0)	104 (18.3)	0.891
Comorbid Conditions			
Number of comorbid conditions, mean (SD)	2.16 (1.36)	1.68 (1.32)	0.006
Comorbidity, *n* (%)	31 (49.2)	202 (35.6)	0.034
Hypertension, *n* (%)	41 (65.1)	307 (54.1)	0.098
Diabetes mellitus, *n* (%)	18 (28.6)	128 (22.6)	0.285
Mental Health			
Depressive symptom (GDS score ≥6), yes, *n* (%)	36 (57.1)	87 (15.3)	<0.001
Physical Activity			
Moderate/vigorous activity, none, *n* (%)	38 (60.3)	185 (32.6)	<0.001
Social Activity			
Religious meeting, none, *n* * (%)	31 (49.2)	217 (38.3)	0.094
Social meeting, none, *n* (%)	19 (30.2)	161 (28.4)	0.769
Possible Malnutrition			
MNA score ≤11, *n* * (%)	14 (22.6)	71.4 (6.2)	<0.001
Self-perceived health, poor, *n* (%)	43 (68.3)	157 (27.7)	<0.001

Notes: Comorbidity: ≥2 comorbid conditions; GDS = Geriatric Depression Scale; MNA = Mini Nutritional Assessment; MMSE = Mini-Mental State Examination. Chi-squared test for categorical variables and independent *t*-test for continuous variables. *n* * indicates missing data.
